# ECG parameters in episodes of neonatal hyper- and hypoglycemia: an observational study

**DOI:** 10.3389/fped.2026.1937246

**Published:** 2026-09-10

**Authors:** Lena Olivier, Julie Strunk, Camelia Lauterbach Oprea, André Stollenwerk, Sina Wedding, Thorsten Orlikowsky, Mark Schoberer

**Affiliations:** 1 Uniklinik RWTH Aachen, Department of Pediatric and Adolescent Medicine, Division of Neonatology, Aachen, Germany; 2 RWTH Aachen University, Chair for Embedded Software (Computer Science 11), Aachen, Germany

**Keywords:** blood glucose, electrocardiography, hyperglycemia, hypoglycemia, neonatal intensive care units, neonate

## Abstract

**Introduction:**

Abnormal glucose concentrations are common in the neonatal period and may entail long-term consequences. They are currently detected invasively through spot-measurements, contribute to blood loss and are painful depending on the sampling route. We hypothesized that hypo- and hyperglycemia may be associated with changes in neonatal electrocardiogram (ECG), such as PR interval, QRS duration, and ST segment durations.

**Methods:**

We analyzed ECG parameters and categorized blood glucose measurements [hypo-, normo-, hyperglycemia (locally defined as >180 mg/dL)] using generalized linear mixed-effects models. Potential ECG predictors of blood glucose abnormalities were first identified in univariable models and then entered into a multivariable model to assess associations, adjusting for postmenstrual age and time between two consecutive measurements. The direction and magnitude of observed associations were assessed in leave-one-subject-out sensitivity analyses.

**Results:**

959 paired measurements (32 hypoglycemia, 714 normoglycemia, 213 hyperglycemia) from 15 neonates were analyzed. In the multivariable model, PR interval was significantly associated with hypo- and hyperglycemia. QRS duration was associated with hyperglycemia, whereas ST segment duration was related to hypoglycemia. All associations remained significant after adjustment for postmenstrual age and time between two consecutive measurements. Within the constraints of the limited sample size, the leave-one-subject-out sensitivity analysis suggested that the direction and magnitude of effect sizes were largely consistent across iterations. However, statistical significance was sensitive to single-subject exclusions, particularly for the PR interval in hypoglycemia. Intraclass correlation coefficients were 0.65 for hyperglycemia and 0.41 for hypoglycemia, indicating relevant intra-individual clustering.

**Discussion:**

The findings of this hypothesis-generating study in 15 individual infants suggest that cardiac electrophysiology may provide information about blood glucose abnormalities in the neonatal period. Especially findings on hypoglycemia remain exploratory due to the limited sample size. ECG parameters may serve as candidate features for non-invasive prediction of hypo- and hyperglycemia in critically ill neonates.

## Introduction

### Background

The maintenance of the blood glucose homeostasis in neonates is essential to support normal brain function, growth, and adaption to extrauterine life. The neonate's brain has a high demand for glucose, while the infant's ability to regulate blood glucose is still developing (1). Abnormalities of blood glucose concentrations (BGC) are an everyday challenge in neonatology. Multiple studies found that more than 50% of premature infants are affected by hyperglycemic episodes (2, 3). Recurrent and prolonged episodes can lead to glucosuria and dehydration and were furthermore found to be associated with intraventricular hemorrhage (2), retinopathy of prematurity, respiratory morbidities (4) and sepsis (5). Contrary, hypoglycemic episodes are less frequent (5-15% of neonates) (6), while the incidence in high-risk groups such as diabetic fetopathy or hypotrophic premature infants was reported to be much higher (up to 50%) (7). Prolonged or repeated hypoglycemia can result in seizures and impairment of the neurodevelopmental outcome (8), while also triggering acute stress responses that disrupt autonomic cardiovascular control and cardiac electrophysiology. Both recurrent and prolonged severe hypo- and hyperglycemia are associated with increased mortality and length of hospital stay (9).

BGC are typically monitored invasively through blood sampling and analysis. This procedure contributes to blood loss, is painful in case of heelstick sampling and is not compatible with minimal handling strategies (10, 11). Less invasive methods to detect abnormal BGC and guide targeted testing would therefore be desirable to decrease patient stress.

For pediatric and adult patients, prior studies investigated the electrocardiogram (ECG)-based detection of abnormal BGC in type 1 diabetes patients (12–15). Multiple ECG parameters were found to be altered during episodes of hypo- and hyperglycemia, e.g., heart rate (HR) and QTc interval.

We hypothesized that neonatal hypo- and hyperglycemia would also be associated with alterations in the ECG.

### Objectives

ECG parameters during episodes of neonatal hypo- and hyperglycemia have so far been underexplored. The objective of this hypothesis-generating work was to explore and compare ECG patterns depending on BGC in the neonatal period.

## Materials and methods

### Study design and setting

The study was conducted as a retrospective observational study on the tertiary care neonatal intensive care unit at Uniklinik RWTH Aachen. Annually, approximately 350 patients (aged 0–17 years) are treated on the ward.

### Participants

Neonates enrolled in the AIx-Neo-Guard project were considered eligible for inclusion. The project has been ongoing since 29 August 2024 and investigates clinical and artificial-intelligence-based approaches to address various aspects of neonatal and pediatric intensive care treatment (16). The study was approved by the local ethics committee (Ethik-Kommission an der Medizinischen Fakultät der RWTH Aachen, approval number 24-213) with waiver for informed consent. As of May 2025, the dataset included high-frequency physiological data from 108 neonates.

Neonates were eligible for inclusion in the present study if they experienced at least one episode of hypoglycemia (<45 mg/dL) or hyperglycemia (>180 mg/dL, based on local clinical intervention thresholds) and had simultaneous ECG recordings available (as of 29 May 2025). Patients were excluded if their parents withdrew consent for study participation or if they had congenital heart defects or relevant cardiac arrhythmias.

### Variables and data sources

BGC, potassium and lactate were measured using an ABL90 FLEX Plus analyzer (Radiometer GmbH, Krefeld, Germany). Measurements were categorized as hypoglycemia (<45 mg/dL) (17, 18), normoglycemia (45–180 mg/dL) or hyperglycemia (>180 mg/dL) (19).

HR and three-lead ECGs were recorded by Philips IntelliVue X2 monitors (Philips GmbH Market DACH, Hamburg, Germany). HR was averaged over 5 minutes to exclude the influence of outliers. Non-interpretable segments (e.g., due to motion artifacts or disconnections) were excluded from the analysis. ECG intervals [P wave duration, PR interval, QRS duration, ST segment duration, T wave duration, QT interval, QTc interval (calculated using Bazett's formula), RR interval] and ST segment characteristics were assessed manually. ST amplitude was measured as the deviation of the J-point from the PR baseline (20). ST depression and elevation were defined as mean ST amplitudes of < −0.1 mV (depression) or > 0.1 mV (elevation) in 20 consecutive heart beats (21–23).

Each ECG parameter was measured in one representative ECG cycle (except for ST depression and elevation). Because approaching the infant and blood sampling has an influence on ECG measurements (mostly through motion artifacts) and the cardiac physiology (through pain-related stress, e.g., tachycardia), we sought the last regular ECG cycle before such disturbances became apparent. This cycle needed to be within a 5 minute window before the timestamp of BGC analysis to ensure representativeness.

Postmenstrual age (PMA), sepsis diagnosis, information on invasive mechanical ventilation and caffeine administration were obtained from the local patient data management system.

### Bias

The same type of measurement device was used for each patient. Study participation did not alter intensive care diagnostics or treatment due to the retrospective design.

### Study size

All eligible patients were included.

### Statistical methods

The study participants contributed varying numbers of measurements. Therefore, multinomial logistic generalized linear mixed models (GLMM) were used to account for the three-level categorical outcome (hypo-, normo-, hyperglycemia). Categorical BGC values were specified as the dependent variable. Normoglycemia was specified as the baseline reference category. Univariable models were fitted, with each continuous ECG parameter entered separately as a fixed effect to identify possible associations with BGC categories. A random intercept was included to account for patient-specific differences. Statistical significance of fixed effects was assessed using Wald tests, with statistical significance set at the two-tailed 0.05-level. ECG variables were entered using their unscaled raw values in milliseconds. Consequently, all reported Odds Ratios correspond to a 1 ms increase in interval duration. Missing data were not imputed.

Because the number of independent observations (patients) was limited, the exploratory multivariable model was subsequently only constructed for parameters significantly associated with BGC in the univariable analyses to reduce model complexity. The multivariable model was additionally adjusted for (i) time between two consecutive measurements, (ii) PMA (iii) time and PMA, (iv) potassium, (v) lactate, (vi) sepsis, (vii) invasive mechanical ventilation, (viii) caffeine administration and (ix) vasopressor therapy.

To assess the significant associations from prior analyses regarding individual subjects, a leave-one-subject-out (LOSO) sensitivity analysis was performed. Given the small sample size (*n* = 15), the LOSO analysis was intended strictly as an exploratory, hypothesis-generating sensitivity analysis rather than a series of confirmatory hypothesis tests. The analysis was repeated iteratively, excluding one subject per iteration. For each LOSO model, the estimated regression coefficient (ß), odds ratio (OR), corresponding 95% confidence interval (CI) and p-value were extracted. Robustness was evaluated by examining the consistency of the direction and the magnitude of the estimated effects, as well as the stability of statistical inference across the iterations. The proportion of significant LOSO models was used descriptively to characterize the stability of findings; and not as an additional inferential test.

All models were fitted using jamovi (version 2.7.31; The jamovi project, Sydney, Australia) with the GAMLj module (version 3.6.5).

## Results

### Participants and descriptive analysis

**Figure 1:**
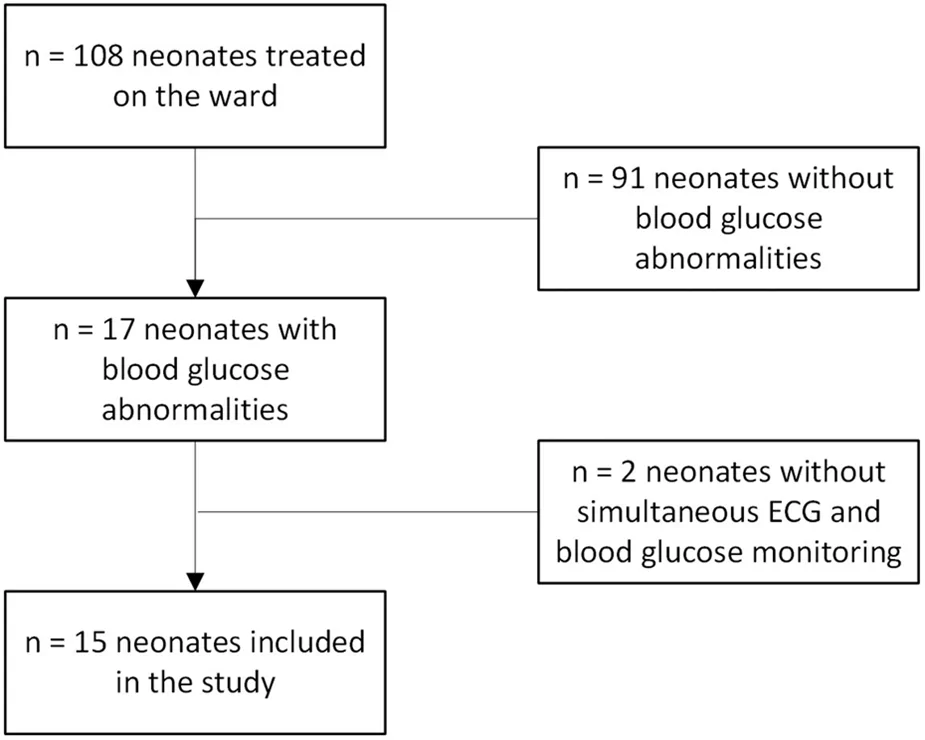
Patient flow chart.

As of 29 May 2025, 108 neonates treated on the ward were included in the AIx-Neo-Guard project. Of these, 17 exhibited BGC abnormalities. 2 patients did not have simultaneous ECG recordings, resulting in 15 patients being included in the analysis (Figure 1). Patient characteristics are shown in Table 1. The study participants contributed a total of 959 measurements. Of these measurements, 32 (3.3%, from 9 infants) were hypoglycemic, 714 (74.5%, from 15 infants) were normoglycemic, and 213 (22.2%, from 10 infants) were hyperglycemic. 5 of 9 patients contributed more than 1 hypoglycemic episode, while 8 of 10 infants exhibited more than 1 hyperglycemic episode. 4 infants contributed both episodes of hypo- and hyperglycemia (Supplementary Table 1). 166 measurements (0 hypoglycemic, 158 normoglycemic, 8 hyperglycemic) from 5 patients were excluded due to non-interpretable (*n* = 52) or missing (*n* = 114) ECG segments. 9 of the non-interpretable ECG segments occurred in septic episodes. Estimated Marginal Mean measurements for the regarded ECG parameters depending on BGC are shown in Table 2.

**Table 1:** Characteristics of the study participants, shown as median [interquartile range] where appropriate.

Patient characteristic	Overall
Number of patients	15
Number of measurements	959
Number of measurements per infant	64 [30; 94]
Male sex (n, %)	10 (66.7%)
Gestational age (weeks)	30 [26; 34]
Birth weight (grams)	1490 [670; 2300]
Postnatal age at measurement	8 [3; 13]
Early onset bacterial infection (n, %)	5 (33.3%)
Invasive mechanical ventilation (n, %)	10 (66.7%)
Non-invasive mechanical ventilation (n; %)	15 (100.0%)
Caffeine administration (n, %)	10 (66.7%)
Maternal diabetes (n, %)	1 (6.7%)
Insulin administration (n; %)	1 (6.7%)
Mild hypertrophic cardiomyopathy (n, %)	1 (6.7%)
Vasopressor therapy (n, %)	6 (40.0%)
Growth status	
- Small for gestational age (n, %)	3 (20.0%)
- Appropriate for gestational age (n, %)	11 (73.3%)
- Large for gestational age (n, %)	1 (6.7%)

**Table 2:** Estimated marginal means (95% confidence intervals)^a^ for ECG parameters across blood glucose categories.

ECG Parameter	Hypoglycemia	Normoglycemia	Hyperglycemia
P wave duration (ms)	53.1 (46.8; 59.4)	51.1 (49.8; 52.4)	48.3 (45.9; 50.7)
PR interval (ms)	86.7 (78.5; 94.9)	93.7 (92.0; 95.4)	90.5 (87.4; 93.5)
QRS duration (ms)	64.2 (55.5; 72.8)	56.3 (54.5; 58.1)	51.4 (48.0; 54.7)
ST segment duration (ms)	54.5 (44.2; 64.8)	31.6 (29.5; 33.7)	35.2 (31.4; 39.0)
ST amplitude (mV)	0.05 (0.02; 0.07)	0.07 (0.06; 0.08)	0.04 (0.03; 0.05)
T wave duration (ms)	145.5 (131.3; 159.7)	162.3 (159.4; 165.2)	142.6 (137.3; 147.8)
QT interval (ms)	263.4 (246.4; 280.5)	250.5 (247; 253.9)	227.8 (221.4; 234.1)
QTc interval (ms)	419.7 (397.7; 441.8)	395.4 (390.9; 399.8)	370.9 (362.7; 379.1)
RR interval (ms)	404.7 (384.8; 424.5)	401.6 (397.5; 405.7)	377.7 (370.1; 385.2)
HR (bpm)	151.5 (145.0; 157.9)	152.6 (151.2; 153.9)	160.8 (158.3; 163.3)

a Values are presented as Estimated Marginal Means (EMM) with 95% Confidence Intervals (95% CI) derived from the linear mixed model (LMM) to account for repeated measures and intra-individual correlation.

### GLMM analyses

**Figure 2:**
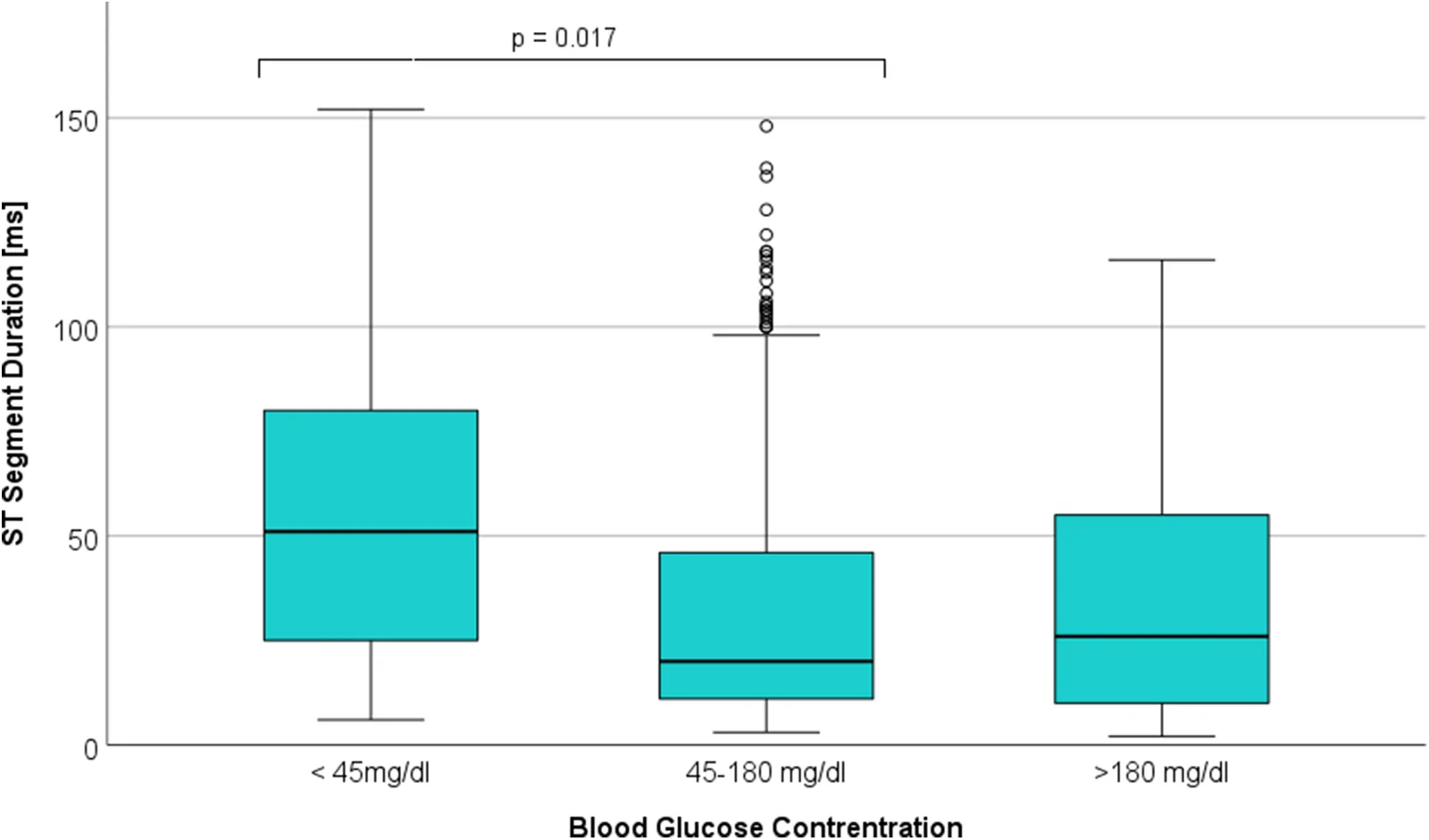
Distribution of ST segment duration according to BGC categories (<45 mg/dL, 45–180 mg/dL, and >180 mg/dL). Statistical comparisons were performed using an multinomial logistic GLMM, with normoglycemia chosen as the reference category.

In the univariable GLMM analyses, several ECG parameters were significantly associated with BGC category (Table 3). P wave duration (OR 1.45, 95% CI 1.09–1.92, *p* = 0.010), QRS duration (OR 1.25, 95% CI 1.05–1.51, *p* = 0.011), ST amplitude (OR 0.68, 95% CI 0.49–0.93, *p* = 0.018), and ST depression (OR 0.24, 95% CI 0.10–0.60, *p* = 0.002) were associated with hyperglycemia, whereas the PR interval showed an association with both hyperglycemia (OR 1.75, 95% CI 1.23–2.49, *p* = 0.002) and hypoglycemia (OR 0.60, 95% CI 0.38–0.96, *p* = 0.034). The ST segment duration was associated with hypoglycemia (Figure 2).

**Table 3:** Univariable GLMMs examining the association between individual predictors and blood glucose category, with normoglycemia chosen as the reference category. Significant associations are highlighted by bold text. Odds ratios are expressed per 1 ms increase in interval duration. CI = confidence interval, OR = odds ratio.

Parameter	Group comparison	Estimate (ß1)	OR	95% CI for OR	*p*-value
P wave duration	Hypo/normo Hyper/normo	−0.18 0.37	0.83 1.45	0.54; 1.29 1.09; 1.92	0.411 **0.010**
PR interval	Hypo/normo Hyper/normo	−0.51 0.56	0.60 1.75	0.38; 0.96 1.23; 2.49	**0.034** **0.002**
QRS duration	Hypo/normo Hyper/normo	0.00 0.23	1.00 1.26	0.61; 1.64 1.05; 1.51	0.998 **0.011**
ST segment duration	Hypo/normo Hyper/normo	0.49 0.08	1.63 1.08	1.17; 2.27 0.83; 1.40	**0.004** 0.573
ST amplitude	Hypo/normo Hyper/normo	−0.48 −0.39	0.62 0.68	0.34; 1.11 0.49; 0.93	0.107 **0.018**
ST elevation	Hypo/normo Hyper/normo	−1.49 0.03	0.22 1.03	0.03; 1.72 0.45; 2.35	0.151 0.941
ST depression	Hypo/normo Hyper/normo	−0.17 −1.41	0.84 0.24	0.17; 4.06 0.10; 0.60	0.831 **0.002**
T wave duration	Hypo/normo Hyper/normo	−0.41 −0.28	0.67 0.76	0.42; 1.05 0.57; 1.01	0.082 0.056
QT interval	Hypo/normo Hyper/normo	0.19 −0.01	1.21 0.99	0.74; 1.96 0.73; 1.34	0.450 0.948
QTc interval	Hypo/normo Hyper/normo	0.32 0.05	1.38 1.05	0.88; 2.15 0.81; 1.37	0.157 0.718
RR interval	Hypo/normo Hyper/normo	0.00 −0.22	1.00 0.80	0.64; 1.56 0.58; 1.10	0.994 0.175
HR	Hypo/normo Hyper/normo	−0.03 0.10	0.97 1.11	0.60; 1.56 0.83; 1.47	0.897 0.483

The significant associations revealed in the univariable models were then incorporated into a multivariable model. PR interval, QRS duration and ST segment duration remained significantly associated with BGC category after adjustment for the other ECG variables. Longer PR interval durations were associated with higher glucose categories, reflected by higher odds of hyperglycemia (OR 2.02, 95% CI 1.18–3.46, *p* = 0.010), and shorter PR interval durations were associated with lower glucose categories (higher odds of hypoglycemia, OR 0.47, 95% CI 0.23–0.97, *p* = 0.041), each compared with normoglycemia. Likewise, longer QRS durations were related to higher odds of hyperglycemia (OR 1.30, 95% CI = 1.05–1.60, *p* = 0.016), whereas longer ST segment durations were associated with higher odds of hypoglycemia (OR 1.52, 95% CI 1.08–2.14, *p* = 0.017). No significant associations were observed between QRS duration and hypoglycemia or between ST segment duration and hyperglycemia.

The associations of P wave duration, ST amplitude, and ST depression observed in the univariable analyses were no longer significant after multivariable adjustment. The results from the multivariable models are presented in Table 4. The intraclass correlation coefficient (ICC) was 0.65 for the hyperglycemia and 0.41 for hypoglycemia, indicating relevant intra-individual clustering.

**Table 4:** Multivariable GLMMs examining the association between multiple predictors and blood glucose category, with normoglycemia chosen as the reference category. Odds ratios are expressed per 1 ms increase in interval duration. Significant associations are highlighted by bold text. CI = confidence interval, OR = odds ratio.

Parameter	Group comparison	Estimate (ß1)	OR	95% CI for OR	*p*-value
P wave duration	Hypo/normo Hyper/normo	0.34 −0.08	1.40 0.92	0.72; 2.75 0.60; 1.42	0.332 0.713
PR interval	Hypo/normo Hyper/normo	−0.74 0.70	0.47 2.02	0.23; 0.97 1.18; 3.46	**0.041** **0.010**
QRS duration	Hypo/normo Hyper/normo	0.01 0.26	1.01 1.30	0.47; 2.15 1.05; 1.60	0.982 **0.016**
ST segment duration	Hypo/normo Hyper/normo	0.42 −0.02	1.52 0.98	1.08; 2.14 0.74; 1.31	**0.017** 0.891
ST amplitude	Hypo/normo Hyper/normo	−0.48 −0.35	0.62 0.71	0.24; 1.57 0.40; 1.26	0.331 0.236
ST depression	Hypo/normo Hyper/normo	1.06 −1.22	2.90 0.29	0.29; 29.41 0.08; 1.09	0.369 0.068

Additional adjustment for time, PMA, time and PMA, potassium, lactate, sepsis, invasive mechanical ventilation or caffeine administration did not materially alter these findings, including the ICC. Adjustment for vasopressor administration (ix) attenuated the association of PR interval and hypoglycemia (OR 0.97, 95% CI 0.94–1.00, *p* = 0.055), suggesting that vasopressors may confound this relationship.

### LOSO sensitivity analysis

Within this cohort, overall consistency in the direction and magnitude of estimated associations was observed across subject exclusions. For all four candidate associations identified as significant in the primary models, the estimated effect direction remained stable across iterations, with a single exception for QRS duration during hyperglycemia in one iteration.

#### PR interval in hyperglycemic episodes

Estimated ORs ranged from 1.027 to 1.038, reflecting a consistently positive association across all LOSO models. The association remained statistically significant in 13 of 15 iterations (86.7%).

#### PR interval in hypoglycemia

All models yielded ORs below 1 (range: 0.962–0.975), suggesting a consistently negative association. However, statistical significance showed higher sensitivity to individual exclusions, with 9 of 15 models (60.0%) reaching *p* < 0.05. In 5 of the 6 non-significant models, p-values remained close to the significance threshold. Despite variation in statistical significance across iterations, the direction and magnitude of the association remained narrow.

#### QRS duration in hyperglycemia

Statistical significance was retained in 14 of 15 LOSO models (93.3%), with ORs consistently of approximately 1.010. In one iteration, the estimated direction reversed (OR = 0.979, 95% CI: 0.949–1.010, *p* = 0.181), suggesting that the subject excluded in this specific model exerted an influential effect on this specific model estimate.

#### ST segment interval in hypoglycemia

Estimated ORs consistently exceeded 1 (range: 1.011–1.017), showing a stable positive association across all iterations. Statistical significance was retained in 13 of 15 models (86.7%).

Overall, within the constraints of this small sample, the LOSO sensitivity analysis suggested that the direction and magnitude of effect sizes were largely consistent across iterations. However, statistical significance proved sensitive to single-subject exclusions, particularly for the PR interval in hypoglycemia. These findings characterize internal cohort stability and highlight associations requiring formal validation in larger, fully powered cohorts.

## Discussion

### Key results

In this hypothesis-generating work conducted on data from 15 individual infants, we identified multiple candidate associations between ECG parameters and abnormal BGC. Specifically, the PR interval was linked to both hypo- and hyperglycemia, with lower BGC corresponding to shorter PR intervals and higher BGC corresponding to longer PR intervals. A longer QRS duration was related to hyperglycemia, whereas longer ST segment durations were observed during hypoglycemia. The direction and magnitude of the observed effects remained consistent in the LOSO sensitivity analyses. Overall, these findings suggest that glycemic deviations in critically ill neonates are linked to measurable ECG interval alterations, with observed patterns driven predominantly by hyperglycemia. Due to the limited number of hypoglycemic episodes, these associations remain exploratory.

### Interpretation

Our findings regarding ST segments and PR intervals are consistent with previous work conducted in pediatric and adult populations. Studies in older patients have repeatedly reported associations between ST segment duration changes and BGC abnormalities (24–26), and we were able to reproduce these observations in our neonatal cohort. One study additionally identified an association of the PR interval with both hypo- and hyperglycemia (26), which aligns with the results of our multivariable model, where PR interval changes were related to both conditions. However, in the neonatal cohort, we could not reproduce previous associations of BGC with QT intervals.

Importantly, prior studies on ECG-based BGC estimation have almost exclusively focused on adolescent and adult patients, leaving neonates systematically under-represented despite their high vulnerability to both hypo- and hyperglycemia. The neonatal period is particularly critical, as recurrent and prolonged glucose disturbances are associated with adverse outcomes (27, 28) and can lead to serious long-term neurodevelopmental and psychological impairment (29–31). Prompt recognition of anomalies and the use of non-invasive approaches promise a particularly high benefit for this patient group. They would allow to conduct more targeted invasive measurements, rather than a replacement for blood glucose testing, and might reduce patient stress (29).

However, glucose disturbances in critically ill neonates typically occur within a broader constellation of metabolic and physiological derangements (e.g., in sepsis) (32). The observed associations might therefore also reflect the overall disease state rather than a direct, isolated effect of BGC (33, 34). Speculatively, underlying cellular mechanisms could play a role. Both hypo- and hyperglycemia might introduce cellular metabolic stress or energy depletion, which could theoretically disrupt cardiac impulse generation and conduction.

Previous studies have demonstrated the feasibility of BGC estimation from ECG parameters using multi-parametric mathematical and artificial intelligence-based approaches (35–37). In a meta-analysis, Liu et al. concluded that current machine learning models can predict adverse glucose levels with reasonable performance (36). The present results may help to refine such models by exploring specific ECG intervals as potential predictors of abnormal BGC in neonates. Future studies should validate these associations prospectively and in larger cohorts, disentangle direct glucose effects from confounding disease processes, and test whether ECG-based algorithms can support earlier, non-invasive detection of hypo- and hyperglycemia in neonatal intensive care.

### Limitations

This explorative analysis was intended to generate hypotheses. It included only a limited number of patients and few episodes of hypoglycemia. Findings regarding hypoglycemia must therefore be regarded as exploratory. Generalizability is therefore limited and external validation is required. Due to the sample size constraints, simultaneous inclusion of all candidate predictors in the multivariable model was not feasible without risking model overfitting. Consequently, we employed a univariable screening step to select variables for the final multivariable GLMM. While this approach is pragmatic under sample size limitations, we acknowledge that univariable pre-selection is subject to potential type I error inflation and may overlook important confounding factors. Therefore, our multivariable findings should also be interpreted as exploratory rather than definitive evidence of independent associations.

Furthermore, we categorized glycemic status into three discrete clinical states (hypoglycemia, normoglycemia, and hyperglycemia). While this aligns with established clinical decision-making thresholds and accounts for non-linear physiological responses, modeling binned outcomes inherently discards continuous dose-response information.

Definitions of hypo- and hyperglycemia are heterogeneous in literature. We defined hyperglycemia as BGC > 180 mg/dL instead of 150 mg/dL, because higher BGC were tolerated during treatment on our neonatal intensive care unit according to local standards. Because BGC were routinely obtained with every blood gas analysis, the measurements reflect different time intervals from feeding, not only fasting BGC.

Regarding ECG monitoring, only one ECG lead (I, II or III) was available. The type of lead was not regarded in this analysis, but could potentially influence the results (especially ST amplitude and depression/elevation, which are lead-dependent). Furthermore, ECG electrode position sometimes deviated from common standards because of the small body size and restrictions through e.g., wound dressings, other sensors and patient positioning. This may affect interval measurements. Amplitudes for parameters other than ST were not considered, because they are dependent on the electrode positions.

Additionally, ECG parameters were assessed manually using a single representative cardiac cycle, whereas heart rate was averaged over a 5-minute period. This introduces two limitations: potential selection bias and inter-observer variability due to the lack of beat-averaging or dual-reader validation, and a temporal discrepancy between short-term morphological measures and longer-term average heart rates. Given the exploratory scope of this study, these preliminary findings warrant future validation using automated, multi-cycle beat-averaging methods.

Within the study cohort, there was only one infant of a diabetic mother, one infant with mild echocardiographic hypertrophy and one infant receiving insulin therapy. Alongside with weight percentiles (small, appropriate, large for gestational age), acid-balance (e.g., pH or base excess) and glucose administration rates, these covariates should be explicitly analyzed in larger study cohorts. Future work is required to address these limitations.

While non-invasive methods for the early detection of abnormal blood glucose levels could ideally guide targeted invasive sampling, they may also increase the overall volume of blood draws due to confirmatory testing or contribute to alarm fatigue.

## Conclusion

In this exploratory, hypothesis-generating work, we identified potential significant associations of ECG parameters with abnormal BGC in the neonatal period. Due to the limited number of hypoglycemic episodes, the observed associations should carefully be interpreted as exploratory. These findings suggest that cardiac electrophysiology may contain useful information on glucose disturbances and could serve as a non-nvasive source of features for future prediction models in this vulnerable population. Given the observational design, limited sample size and the frequent coexistence of broader metabolic and physiological derangements, the observed associations should carefully be interpreted as hypothesis-generating and exploratory. Prospective studies on larger cohorts are needed to evaluate their reproducibility and examine possible other parameters (e.g., T wave sharpness), and to determine whether ECG-based algorithms can support earlier, clinically meaningful detection of BGC abnormalities in neonatal intensive care.

## Data Availability

The data analyzed in this study is subject to the following licenses/restrictions: Data may only be accessed for predefined research questions due to data protection regulations. Requests to access these datasets should be directed to Lena Olivier, lolivier@ukaachen.de.
