# Supplementary material for: ECG parameters in episodes of neonatal hyper- and hypoglycemia: an observational study
Source: Front Pediatr. 2026 Sep 10;14:1937246. doi: 10.3389/fped.2026.1937246 (PMC13601268; doi:10.3389/fped.2026.1937246)
Supplement: Supplementary file 1 [file Table1.docx]

**Supplemental Table 1:** Distribution of measurements among the study cohort (shown as absolute number and percentage)

| Patient | Number of measurements | Hypoglycemia (<45 mg/dl) | Normoglycemia (45-180 mg/dl) | Hyperglycemia (>180 mg/dl) |
| --- | --- | --- | --- | --- |
| 1 | 133 | 0 (0.0%) | 132 (99.2%) | 1 (0.8%) |
| 2 | 74 | 2 (2.7%) | 67 (90.5%) | 5 (6.8%) |
| 3 | 118 | 0 (0.0%) | 99 (83.9%) | 19 (16.1%) |
| 4 | 22 | 1 (4.5%) | 21 (95.5%) | 0 (0.0%) |
| 5 | 39 | 0 (0.0%) | 38 (97.4%) | 1 (2.6%) |
| 6 | 49 | 0 (0.0%) | 46 (93.9%) | 3 (6.1%) |
| 7 | 100 | 0 (0.0%) | 6 (6.0%) | 94 (94%) |
| 8 | 89 | 4 (4.5%) | 40 (44.9%) | 45 (50.6%) |
| 9 | 74 | 0 (0.0%) | 65 (87.8%) | 9 (12.2%) |
| 10 | 94 | 10 (10.6%) | 82 (87.8%) | 2 (2.1%) |
| 11 | 8 | 1 (12.5%) | 7 (87.5%) | 0 (0.0%) |
| 12 | 64 | 7 (10.9%) | 57 (89.1%) | 0 (0.0%) |
| 13 | 30 | 2 (6.7%) | 28 (93.3%) | 0 (0.0%) |
| 14 | 5 | 1 (20.0%) | 4 (80.0%) | 0 (0.0%) |
| 15 | 60 | 4 (6.7%) | 22 (36.7%) | 34 (56.7%) |
